# Doppler optical coherence tomography for energy seal evaluation and comparison to visual evaluation

**DOI:** 10.1117/1.JBO.25.3.035003

**Published:** 2020-03-09

**Authors:** Andrew J. Marques, Robnier Reyes, Christopher R. Pasarikovski, Chaoliang Chen, Joel Ramjist, Xijia Gu, Victor Yang

**Affiliations:** a Ryerson University, Bioengineering and Biophotonics Laboratory, Department of Electrical, Computer, and Biomedical Engineering, Toronto, Ontario, Canada; b University of Toronto, Division of Neurosurgery, Department of Surgery, Toronto, Ontario, Canada; c Ryerson University, Department of Electrical, Computer, and Biomedical Engineering, Toronto, Ontario, Canada; d Sunnybrook Health and Sciences Center, Division of Neurosurgery, Toronto, Ontario, Canada; e University of Toronto, Division of Neurosurgery, Faculty of Medicine, Toronto Ontario, Canada

**Keywords:** energy sealing, optical coherence tomography, hemostasis, thulium

## Abstract

Laser energy sealing systems have attracted much attention over the past decade given the general shift in surgical paradigm toward less invasive surgical approaches. Given this, it is paramount to have an objective method with which the quality of energy seals can be evaluated. Current methodologies used for this purpose can be problematic in the evaluation of small vessel seals. A methodology employing Doppler optical coherence tomography (DOCT) for the evaluation of energy seals is introduced. Avian chorioallantoic membrane vessels were subjected to thulium laser irradiation and were then scanned via OCT. Outcomes were classified based on several markers, predominantly the presence or absence of flow postirradiation. Vessel diameter and general morphology were also taken into consideration. Vessels were classified into four groups: seal (29%), rupture (30%), partial seal (19%), and unaffected (22%). All vessels were also evaluated visually by a trained neurovascular surgeon, and these visually classified outcomes were compared with DOCT evaluated outcomes. It was found that whether the vessel was considered sealed or not sealed was dependent on the evaluation method (p=0.01) where visual classification resulted in 18% more seals than DOCT classification. Further, the specificity of visual classification was found to be strongly dependent on the number of partial seals (p<0.0001). DOCT has shown to be an indispensable method for the evaluation of energy seals not only solely due to its high velocity resolution but also due to valuable microscopic morphological insight regarding the biological mechanisms responsible for energy sealing.

## 1 Introduction

Laser energy sealing is the process by which a laser is used to occlude a blood vessel. It has numerous applications across multiple surgical sectors including tumor devascularization,^1^^,^^2^ the treatment of various endolaryngeal maladies,^3^^–^^5^ and the treatment of arteriovenous malformations.^6^^,^^7^ In all cases, the intent is to mitigate the flow of blood to a body of tissue with minimal collateral damage or simply to achieve hemostasis by ceasing the extravasation of blood. Laser-based systems are an attractive option for the energy sealing of vessels as they offer several advantages over conventional instruments. These include the potential to induce hemostasis in a contactless fashion and the ability to achieve selective tissue interaction with wavelength selection.^8^ In the last decade or so, these systems have attracted more attention, given the general shift in surgical paradigm toward the use of less invasive surgical instruments and the general ease with which laser light can be conducted through such instruments. Given this, it is paramount to have an objective method with which vessel seals can be evaluated so that the hemostatic capabilities of laser-based systems can be tested. Current methodologies used for this purpose include burst pressure analysis (BPA),^9^^–^^11^ microscopy,^12^ and visual examination.^13^^–^^16^ While BPA yields valuable insight as to the strength of seals, it cannot be performed *in situ* without invasive measures. Microscopic analysis allows for detailed views of the irradiation site and is effective in observing blood flow even in small arteries. However, it relies on an unimpeded view of the vessel, which at times is not granted, given the development of coagulum, and further does not yield depth-resolved information. It is crucial to evaluate the entirety of a vessel cross-section for flow in order to ensure that a vessel has been completely sealed, thus depth-resolved information is needed. Visual examination is a rapid method and is ideal in noncritical scenarios where complete cessation of blood flow is not necessary. Despite this, it is highly subjective, making it a poor choice for scientific processes and impractical for vessels below 1 mm in diameter due to limitations of the human eye.

In this paper, a new modality for the evaluation of laser energy seals is introduced: Doppler optical coherence tomography (DOCT). DOCT imaging features a high sensitivity to flow, typically capable of detecting flows as low as 130  μm/s in tissue.^17^ Clinical applications of DOCT include vasoactive drug screening, hemodynamic monitoring following pharmacological and photodynamic therapy, and mapping cortical hemodynamics.^18^ In a study comparing Doppler ultrasound to CT angiography, the gold standard in vascular flow detection, it was found that Doppler ultrasound correctly detected 50/58 carotid occlusions.^19^ Of the eight false positives (no flow detection when in fact there was flow), seven were cases of near complete occlusion, thus extremely low flow rates, and one was a result of an anatomical anomaly where a sharp 90 deg bend of the vessel occurred in the imaging field resulting in failed Doppler signal acquisition. DOCT has two orders greater velocity resolution than conventional Doppler ultrasound systems^17^ and thus, it is likely to succeed in detecting flows present in near totally occluded vessels. As such, it is reasonable to conclude that DOCT will have a higher sensitivity (i.e., in detecting complete seals) than Doppler ultrasound bringing it closer to the gold standard of CT angiography. The primary limitation of DOCT is its shallow penetration depth, which limits the depth at which velocity information can be resolved to a maximum of 3 mm. This means that DOCT, for the purposes of energy seal evaluation, is only viable for small vessels with diameters below 3 mm. Further, DOCT can only target superficial vessels, vessels that have been surgically exposed, or vessels that have been brought within close proximity of the instrument in laparoscopic/catheter-based cases. Despite this, DOCT offers an objective method for evaluating the quality of energy seals and given its high velocity resolution is capable of detecting minute flows that are otherwise invisible to the eye. Although DOCT does not generate information relating to the strength of energy seals, as with BPA, its potential for rapid *in situ* assessment of energy seals in small vessels makes it an attractive method for the evaluation of laser sealing systems.

The purpose of this study is to develop a methodology for the evaluation of energy seals induced in small vessels using DOCT. The intent is to use this methodology to evaluate the hemostatic potential of laser-based systems in an objective manner. To the author’s knowledge, there have been no studies that employ DOCT for this purpose. In the literature, several studies exist where a laser’s ability to induce complete seals in vessels is evaluated visually. Therefore, given the nature of this study, it is valuable to compare visual evaluation with DOCT evaluation such that the accuracy of visual methods in detecting blood flow can be assessed.

## 2 System Description

**Fig. 1:**
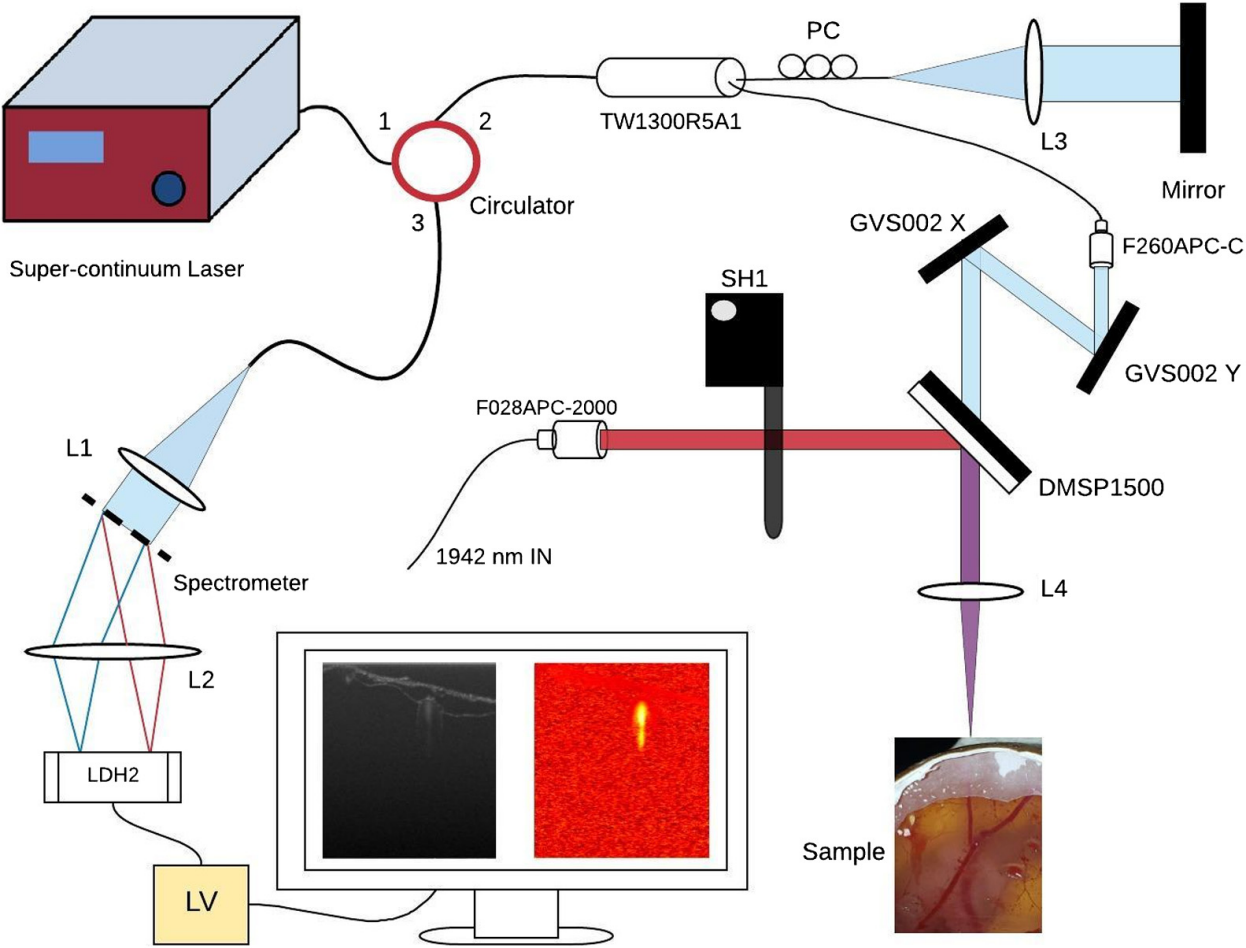
Optical system diagram. L1-L4, achromatic lenses. A polarization controller is situated in the reference arm of the interferometer (PC). LV, LabVIEW processing block.

A thulium fiber laser with 1942-nm output was used in this experiment to seal vessels. It has been reported previously.^20^ The thulium fiber laser is compact, high-powered, tuneable, and lases at a strongly hydrolytic wavelength and thus is especially well suited to medical applications. As can be seen in Fig. 1, imaging was accomplished using a spectral-domain optical coherence tomography (OCT) system with a supercontinuum source (SuperK Extreme from NKT Photonics) centered at 1310 nm. In medical systems, it is important to minimize cost, thus, although a swept source system may have offered better performance,^21^ a spectrometer-based system was employed instead. The fiber-based Michelson interferometer consists of a THORLABS CIR-1310-50-APC circulator and a THORLABS TW1300R5A1 beam-splitter to maintain the wide bandwidth of the supercontinuum source. It also includes a three-paddle polarization controller to facilitate polarization matching. At the sample arm, the beam is collimated using the THORLABS F260APC-C collimation package before passing through the GVS002 galvo system for sweeping. The beam was swept in one-dimension over a distance (at the sample) of 6.2 mm. Power balance between the reference and sample arms was carefully done during the calibration phase. Dispersion compensation between the arms was done numerically in software. The interference signal was acquired using a custom spectrometer from P&P Optica, which has a grating frequency of 892  lines/mm, a spectral resolution of 0.365 nm, and a wavelength range of 1190 to 1370 nm, achieving an axial resolution of ∼6  μm in tissue. This in comparison with the resolution of high-frequency ultrasound, which is typically between 50 and 100  μm.^22^ The camera of the spectrometer (Goodrich LDH2) contains an array of 1024 sensors being sampled at 10,813 Hz (A-line rate). The sampled signal was processed using a LabVIEW VI through the steps of resampling, FFT, dispersion compensation, and log compression for display. A short-pass dichroic mirror (THORLABS DMSP1500) was used to couple the 1942- and 1300-nm beams. The mirror was fixed at exactly 45 deg using the THORLABS C3P60R. After passing through the mirror, the coupled beam was focused using a ${\mathrm{CaF}}_{2}$ lens (f=100  mm). Using a beam profiler, the resulting spot size at focus of the OCT beam was found to be ∼70  μm. At 6.2-mm lateral beam sweep and 1024-A-lines per sweep, this ensured sufficient overlap for the acquisition of Doppler signals. Profilers in the 2-μm regime are extremely costly thus spot size of the 1942-nm beam was approximated theoretically using

$$2w_{0}=\frac{4\lambda f}{\pi d},$$ (1)

where $2w_{0}$ is the diameter of the spot at the sample, λ is the wavelength, f is the focal length, and d is the diameter of the spot at the focal lens.^23^ The minimum spot size at the sample was approximated to 206  μm where d=1.2  mm. In this work, the effects of spot size on the seal outcome were not considered. The spot size was varied such that a variety of laser irradiation outcomes could be observed. For this reason, a theoretical value for the spot size was sufficient. The velocity resolution of the DOCT system was found to be 112  μm/s. For comparison, the minimum detectable blood flow velocity of a 6-MHz Doppler ultrasound system is typically 6.4  mm/s;^24^ however, velocity resolutions of ∼100  μm/s have been reported in higher frequency systems.^25^ The velocity resolution of the DOCT system used here was calculated using the minimum detectable phase shift corresponding to an SNR of 25 in the following equation:

$$V=\frac{\lambda_{0}\Delta \varphi }{4\pi \Delta t\;\cos\;\theta },$$ (2)

where V is the velocity resolution, Δφ is the minimum detectable phase shift, Δt is the A-line interval, θ is the Doppler angle (taken to be 180 deg for velocity resolution), and $\lambda_{0}$ is the central wavelength of the OCT system. The SNR was obtained using methods outlined in Ref. 26. For reference, typical blood flow rates in chorioallantoic membrane (CAM) vessels ranging from 100 to 300  μm in diameter have been measured to be between 1 and 8  mm/s,^27^^,^^28^ whereas blood flow rates in human vessels ranging from 0.8 to 1.8 mm in diameter were measured to be between 4.9 and 19.0  cm/s in arteries and 1.5 and 7.1  cm/s in veins.^29^

The entire optical payload, consisting of both high-power laser optics and OCT optics, is mounted onto a linear translation stage and a custom-built pitch and yaw stage, which was necessary due to the high angular sensitivity of the dichroic mirror. Exposure time was regulated using a THORLABS SC1 shutter controller and an SH1 single blade shutter, which was placed between the 1942 nm collimator and the dichroic mirror. Bulk alignment between the imaging and high-powered beams was accomplished using visible lasers. Alignment was verified at the start of each experiment by ablating moistened cardboard and ensuring that the resultant crater was within the imaging field of the 1300-nm beam using real-time OCT structural image feedback.

## 3 Methodology

All experiments were carried out according to recommendations provided by the research ethics board of the institution. A total of 80 avian egg embryos were cultivated and appropriately prepared for the experiment, which yielded 104 viable vessels. Each vessel was subjected to thulium laser irradiation, where exposure time, spot size, and average power were varied in order to generate a variety of outcomes. For each vessel, an OCT structural image, OCT Doppler image, and photograph were acquired for both pre- and postlaser exposure forming a single data point. Pre- and postimages were examined to evaluate the outcome.

### 3.1 *In-Ovo* Specimen Cultivation

**Fig. 2:**
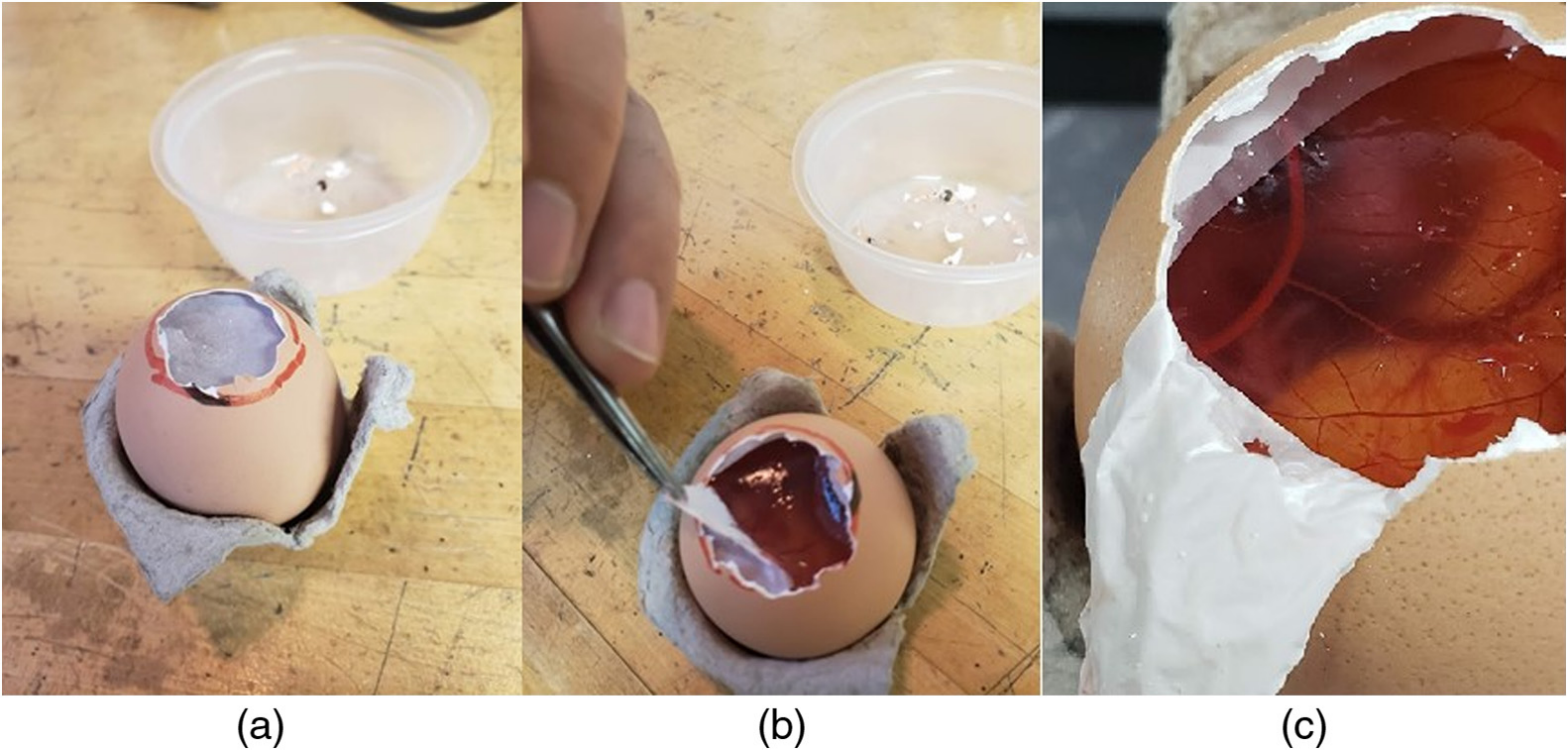
Depiction of specimen preparation. (a) Egg shell removed in the viscinity of the airpocket exposing inner shell membrane. (b) Inner shell membrane separation from CAM. (c) Inner shell membrane shown separated from CAM.

Avian embryos were used in this study as they offer convenient access to functioning arteries and are a rapid, cost-efficient alternative to other *in vivo* models. Eggs were incubated in a custom-built incubator. Access to embryonic arteries was achieved using a method outlined in Ref. 14 where the eggshell is breached in the vicinity of the air-pocket as to avoid damaging vital biological structures. Vessels were exposed by delicately peeling away the inner shell membrane from the CAM (Fig. 2).

In several preliminary experiments, avian embryos were allowed to develop between 5 and 18 days in order to determine the ideal age for Doppler imaging of CAM vessels. It was found that the optimal age for producing the highest number of viable vessels was between 10 and 12 days, where vessels were deemed viable if they were visible to the naked eye, embedded in the CAM, and small enough to be mostly within the imaging field of the OCT system. Embryos younger than 7 days were underdeveloped; vessels in the CAM membrane were typically not visible to the eye and air-pockets were not large enough to provide a sufficient working area. While 15-day old embryos produced many viable vessels, at this age embryos were well developed and tended to produce bulk motion artifacts in the Doppler signals.

### 3.2 Experimental Procedure

For each data point, a vessel was first photographed and scanned via OCT where ∼100 B-scans were acquired at 6 B-scans/s. B-scans were appropriately processed to generate both structural and Doppler images. Given that the heart rate of the avian embryo is between 2 and 4 Hz^30^ averaging was used in the processing of both OCT modalities, which ensured that flow variation due to systole and diastole would be filtered. The chosen vessel was targeted using a real-time OCT structural image and was subsequently irradiated with the 2-μm laser. A postirradiation OCT scan was then conducted in an identical manner to the pre-irradiation scan and a photograph was acquired. Great care was taken to ensure that both pre- and post-OCT scans were acquired in the same cross-section. OCT evaluation of outcomes was performed by contrasting pre- and postirradiation OCT images (both Doppler and structural) and thus is abbreviated as DS-Eval. Several markers were of interest primarily the presence or absence of flow in the region of interest (ROI) as demarcated by the accompanying OCT structural image in the post-DOCT image. The ROI used for DS-Eval was the vessel lumen and the immediate area about the vessel including the layer of the CAM superficial to the vessel. In addition to this, markers such as vessel diameter and general morphology were taken into consideration. Visual evaluation (V-Eval) was performed by a trained neurovascular surgeon (C.R.P.) by examining only pre- and postirradiation photographs. A vessel was deemed visually sealed when there was no evidence of arterial luminal filling beyond the coagulated region. This also included arterial vessel extravasation. V-Eval was done independently (i.e., the investigator was blinded from the corresponding OCT images).

### 3.3 Statistical Analysis

The chi-squared statistical test of independence was used to determine if relationships between categorical data exist. Our null hypothesis is that no relationship exists between the variables of interest and that samples are independent. Our specific aim was to determine if coagulation status of the vessel (seal or no seal) after irradiation was related to the method of evaluation (DS or V). The null hypothesis that seal status is independent of the method of evaluation was tested at the 5% level.

## 4 Results

Outcomes were classified by DS-Eval into four groups: seal, rupture, partial seal, and unaffected. Each outcome was classified according to the presence of primary and secondary biomarkers. The presence of the primary marker was absolutely necessary to classify an outcome while the presence of secondary markers served to refute or support the conclusions drawn from the primary marker. Table 1 shows a summary of markers used to classify vessel seals by DS-Eval.

**Table 1:** Summary of markers used for DS-Eval.

Classification	Structural OCT	Doppler OCT
Seal	Evidence of embolism in the post-ROI	No signal present in the post-ROI^a^
	Significant change in vessel morphology from pre- to post-ROI	
Partial seal	Vessel constriction	Signal area reduced by 15% or more between pre- and post-ROIs^a^
	Evidence of partial embolism in postimage	
Unaffected	Vessel morphology mostly preserved between pre- and post-ROIs^a^	Less than 15% change in signal area between pre- and post-ROIs
Rupture	Vessel disappears in postimage (annihilated)^a^ OR	Doppler signal is present at a vessel cross-section adjacent to the irradiation site
	Vessel is not intact, evidence of wall disruption^a^	
	Significant morphological change	Diffuse Doppler pattern present

a Denotes the primary marker.

The mean vessel diameter was 0.50±0.16  mm. Of the 104 vessels irradiated in this study 30 were seals, 31 ruptured, 20 were partial seals, and 23 were unaffected by DS-Eval. In order to compare DS-Eval with V-Eval, the four groups generated by DS-Eval were generalized into seal and nonseal groups where the nonseal group included partial seals, ruptures, and unaffected outcomes. In this way, DS-Eval reported that 29% of vessels had sealed (30 seals and 74 nonseals) compared with V-Eval, which reported that 47% of vessels had sealed (49 seals and 55 nonseals). A true positive (TP) occurred when both DS and V-Eval resulted in a seal, a true negative (TN) occurred when both DS and V-Eval resulted in a nonseal, a false positive (FP) was declared when V-Eval resulted in a seal and DS-Eval resulted in a nonseal, and a false negative (FN) occurred when V-Eval resulted in a nonseal and DS-Eval resulted in a seal. Following this, V-Eval generated 27 TPs, 52 TNs, 22 FPs, and 3 FNs resulting in a sensitivity of 90%, specificity of 70%, and an overall accuracy of 76%. It was found that whether the outcome was classified as sealed or notsealed was dependent on the classification methodology, and this was statistically significant (p=0.01 using a two-tailed chi-squared test with Yates correction). Table 2 gives the contingency table from which the chi-squared statistic was calculated. Further, it was found that V-Eval specificity was dependent on the type of nonseal classification (partial seal, rupture, or unaffected) and this finding was also significant (p<0.0001, chi-squared test). Table 3 shows the contingency table from which the chi-squared value was calculated.

**Table 2:** The association between seal evaluation method and outcomes.

	DS-Eval	V-Eval	Total
Seals	30	49	79
Nonseals	74	55	129
Total	104	104	208

**Table 3:** The association between V-Evals and DS-Eval nonseal groups.

	V seals	V nonseals	Total
Rupture	4	27	31
Partial seals	14	6	20
Unaffected	4	19	23
Total	22	52	74

### 4.1 Detailed Analysis of Biomarkers Used for DS-Eval

**Fig. 3:**
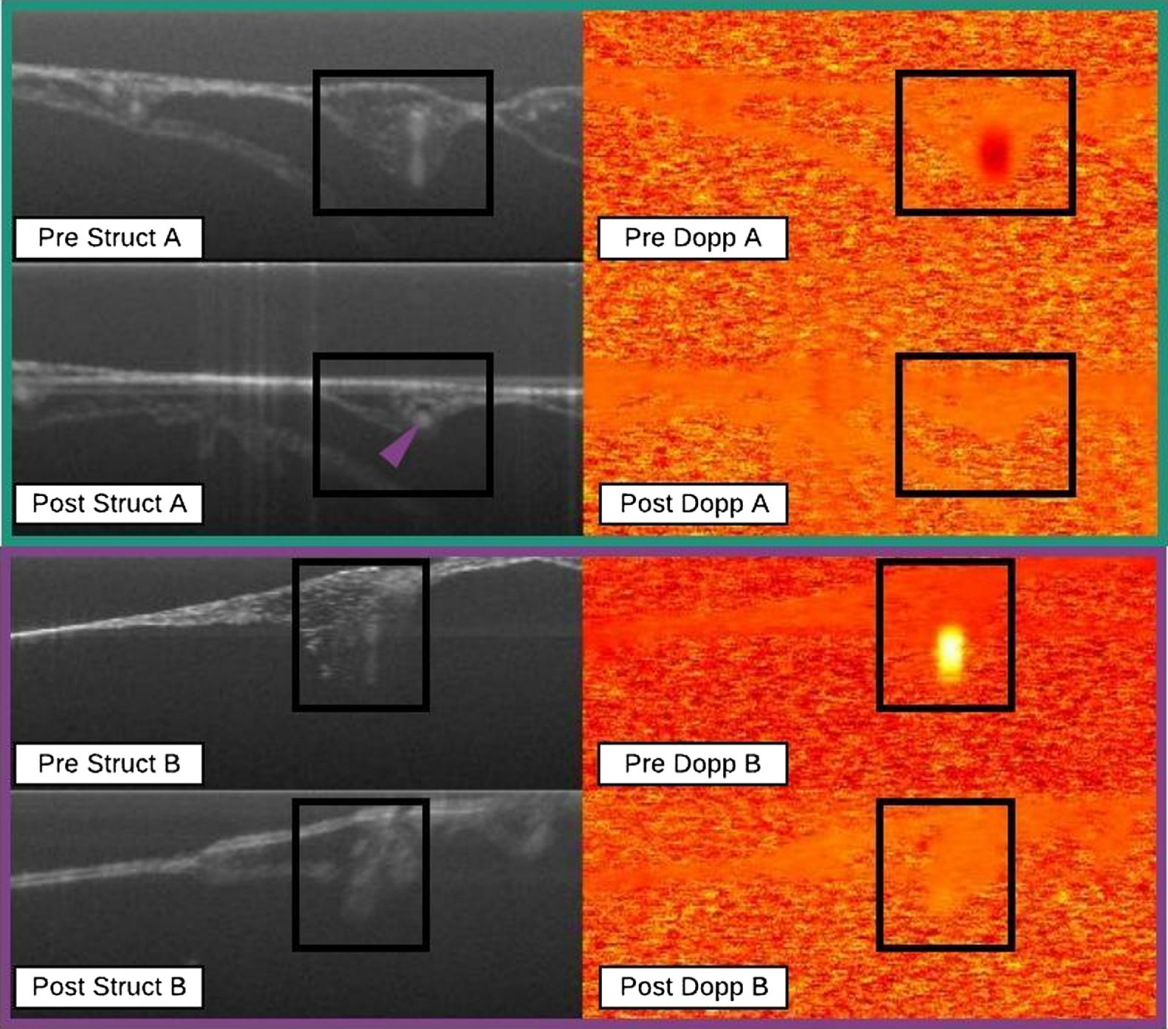
Seal data points A and B. Evidence of embolism (green rectangle) and significant morphological change (purple rectangle). Black boxes represent ROIs and the purple triangle indicates uniform structural signal within vessel lumen suggesting embolism. In all data points note the disappearance of the lemniscate morphology from pre- to poststructural images as well as the disappearance of the Doppler signal in the post-ROI.

Referring to Table 1, the primary marker used for classifying an outcome as a seal was the lack of a Doppler signal in the post-ROI. This was the case for nearly all outcomes that were classified as seals. There were some cases where although the Doppler signal had disappeared in the post-ROI, the outcome was not classified as sealed given that secondary markers strongly indicated otherwise. An unexposed vessel was well characterized by a characteristic lemniscate visible in pre-irradiation ROI. This lemniscate has been detected in other studies using OCT on CAM vasculature.^31^ Thus, if such was the case where the Doppler signal disappeared, and the lemniscate morphology was retained, then the vessel would not be considered sealed. Completely sealed vessels both lacked a Doppler signal in the post-ROI and exhibited a significant change in morphology. A significant change in morphology was considered to have occurred if the characteristic lemniscate disappeared from pre- to poststructural ROI indicating that sufficient energy was delivered to morph the vessel lumen. The strongest structural marker for a seal was evidence of embolism. Embolism was identified by the recognition of a uniform structural signal apparent in the post-ROI, i.e., the characteristic lemniscate morphology was replaced by a homogeneous mass in the vessel lumen. The appearance of this structural marker and the accompanying lack of Doppler signal within the post-ROI indicates that a static structure has developed within the lumen thereby strongly suggesting embolism. Figure 3 gives examples of seal data points that exhibit both evidence of embolism and significant morphological change.

**Fig. 4:**
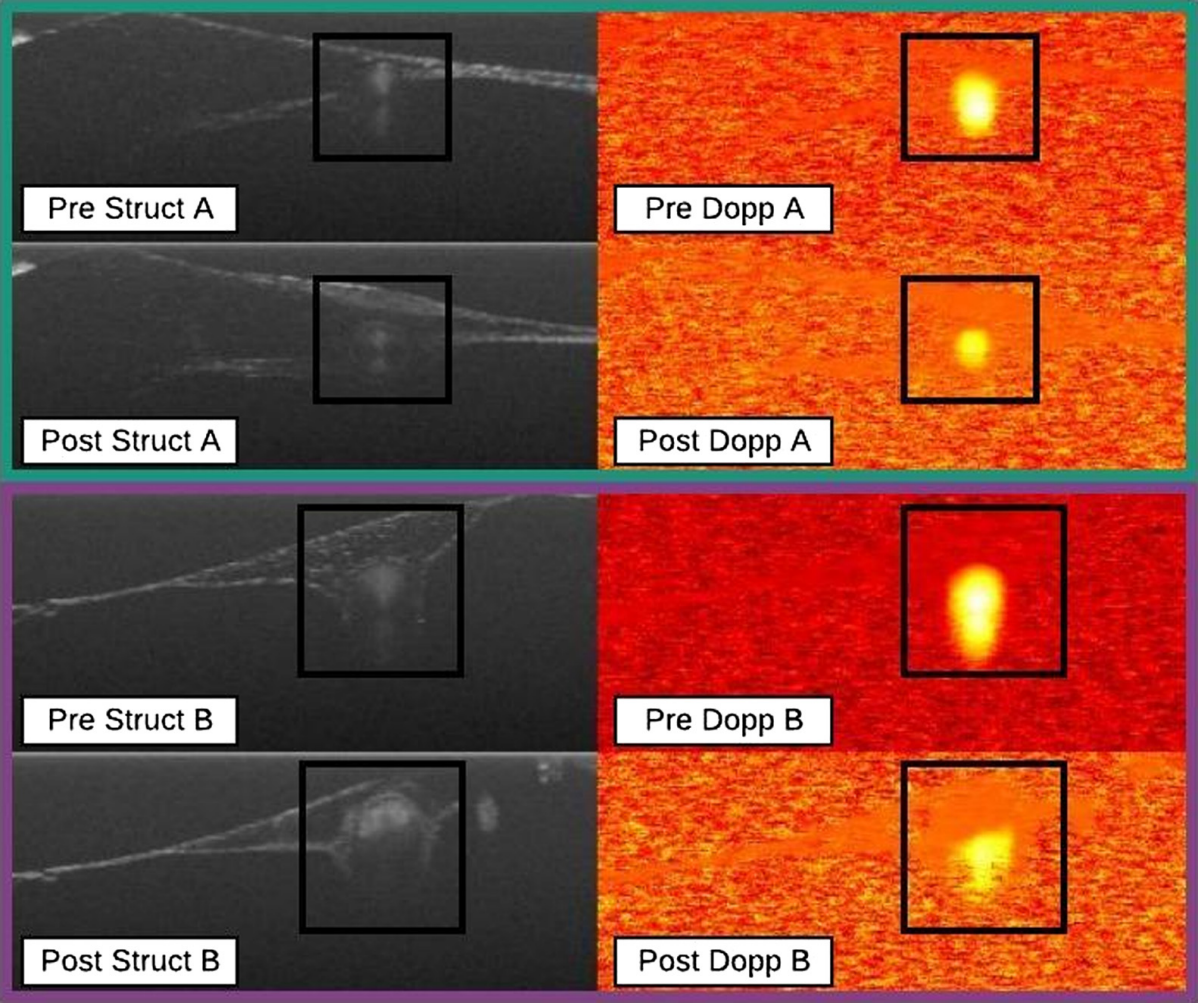
Partial seal data points. Vessel constriction (A, green rectangle) and partial embolism (B, purple rectangle). Black boxes indicate the ROIs. In data point A, the Doppler signal area has clearly been reduced by more than 15% while vessel constriction is apparent from the change in vessel diameter from pre- to poststructural images. In data point B, the Doppler signal area has decreased by more than 15% while from the poststructural image, the homogeneous signal coming from the superficial portion of the vessel lumen accompanied by a lack of a Doppler signal indicates that partial embolism has been achieved.

Partial seals were well defined by a decrease in Doppler signal area between pre- and postirradiation ROIs. To elucidate, a decrease in Doppler signal area meant that the number of pixels demonstrating Doppler signal in the ROI would decrease from pre- to post-Doppler images. In order to differentiate from the unaffected classification, at least a 15% decrease in Doppler signal area must be observed for an outcome to be classified as a partial seal. A change in the Doppler signal area directly indicates that some energy reached the lumen and affected blood flow. In some partial seals, the decrease in Doppler signal area was a result of vessel constriction induced by irradiation. Other cases involved partial embolism, where typically blood had coagulated in the most superficial portion of the vessel but not in the deeper portion. Figure 4 shows both vessel constriction and partial embolism.

**Fig. 5:**
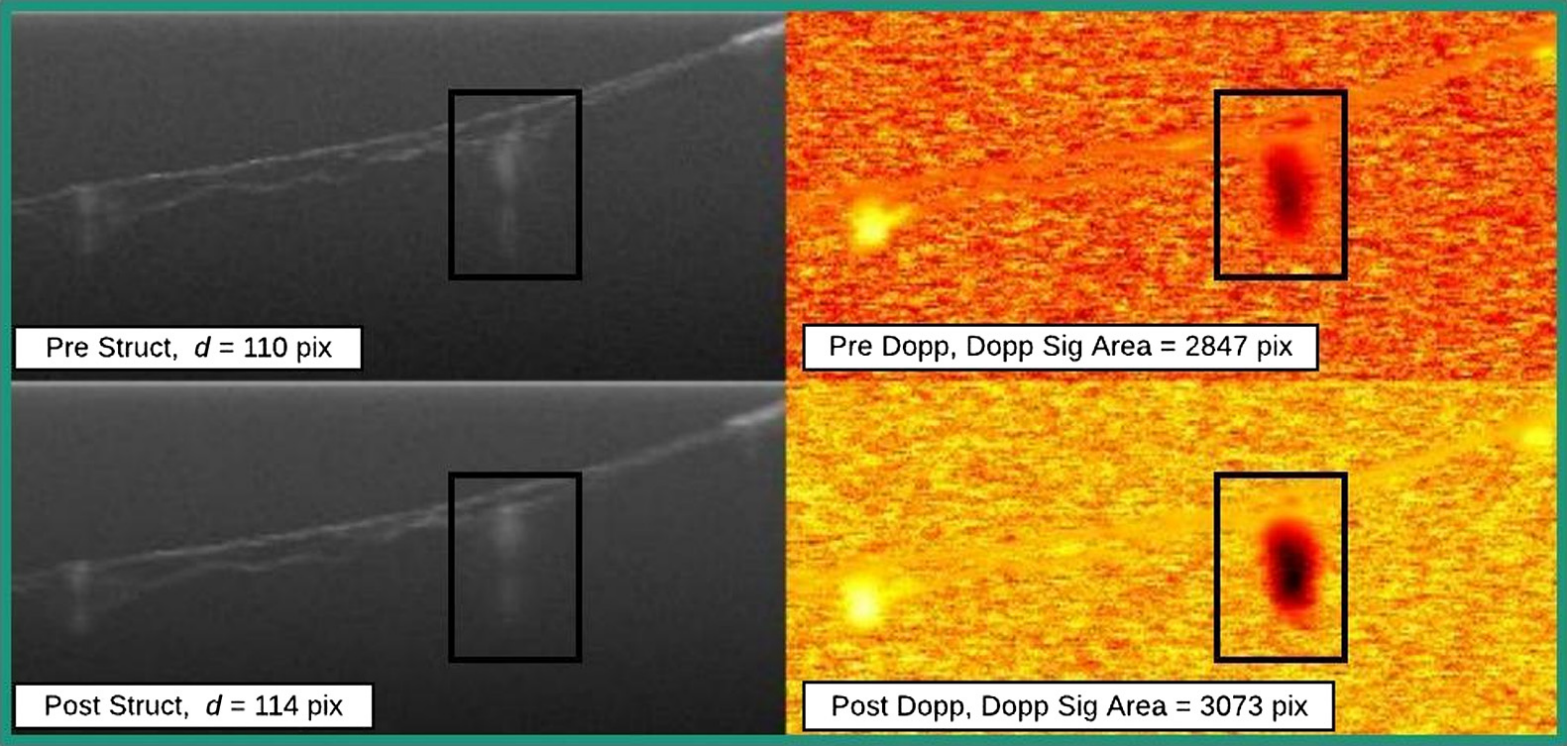
An unaffected data point. Black boxes indicate the ROIs. This data point exhibits <10% change in vessel diameter and <15% change in Doppler signal area and thus is classified as unaffected. Vessel diameters, d, are labeled in each structural image and are reported in pixels. Doppler signal area (Dopp Sig Area) is labeled in each Doppler image and is also given in pixels.

The unaffected classification is a misnomer as many vessel irradiation outcomes in this category certainly exhibited some response to laser irradiation. A more appropriate title for this classification would have been “no significant hemostatic effect” but, for brevities sake, unaffected was used. A vessel was considered unaffected if vessel morphology was mostly preserved from pre- to postirradiation. This was apparent by comparing pre- and poststructural images. “Mostly preserved” indicates that the vessel diameter remained within 10% of its unexposed state and that the lemniscate morphology was present in the poststructural ROI. In addition, for an outcome to be classified as unaffected, the Doppler signal area would have to remain within 15% of its original size. Both the 10% limitation on diameter change and the 15% limitation on Doppler signal area change were arbitrarily chosen to distinguish between unaffected and partial seal outcomes. Here, the primary purpose of DS-Eval was to objectively classify outcomes as sealed or not sealed and thus, since both partial seal and unaffected are both nonseal outcomes, it is not crucial to have objective differentiation between the two categories. The thresholds delineating the two classifications may change to suit the specific needs of a given application of DS-Eval. Figure 5 gives an example of an unaffected data point.

**Fig. 6:**
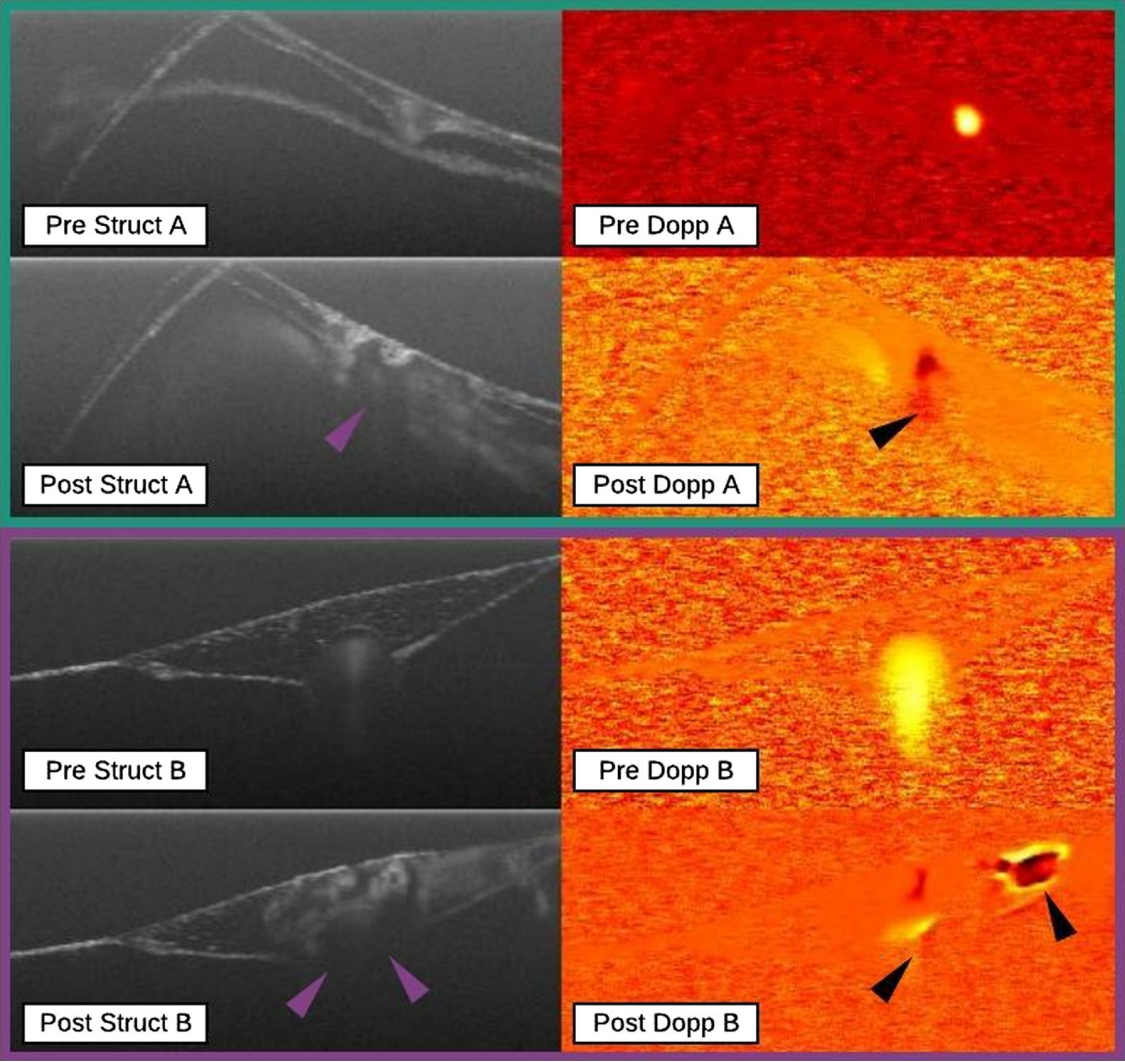
Rupture data points. Both data points A (green rectangle) and B (purple rectangle) demonstrate wall breach indicated by purple arrows while the presence of the diffuse Doppler signal (black arrows) supports the rupture classification. ROIs are left unlabeled for clarity.

Vessel rupture was declared whenever there was evidence of bleeding. Several markers were good indicators of bleeding especially vessel eradication, which was evident from structural images when the vessel was no longer within the field of view. Alternatively, bleeding was also evident when the structural images exhibited vessel wall disruption differentiated from complete annihilation as part of the vessel wall remained intact postirradiation. Secondary markers included the adjacent Doppler signal and the diffuse Doppler signal. The adjacent Doppler signal was acquired at an intact cross section of vessel directly beside the irradiation site. If Doppler signal was present here it was assumed that blood flow had not been ceased within the vessel and thus a rupture was probable. The diffuse Doppler signal was recognized when Doppler signal existed outside of the ROI indicating vascular extravasation. It is termed “diffuse” as the Doppler signal would often be spread over the image and not localized to the ROI. Figure 6 shows two rupture data points with labeled biomarkers.

## 5 Discussion

The primary objective of this study was to introduce Doppler and structural OCT for the evaluation of energy seals induced in small vessels by laser irradiation. The methodology developed termed DS-Eval uses specific biological markers listed in Table 1 to determine whether a vessel had been sealed. In general, the presence of these markers is not based on subjective opinion but rather on numerical information or clear imagery. Incidentally, DS-Eval is objective making it suitable for scientific processes and generating reliable results in the evaluation of laser sealing systems. Acquisition time of each data point was ∼15  min because the specimen had to be maneuvered into the imaging field of the OCT beam and a total of ∼200 B-scans (including pre- and postscans) were acquired at 6 B-scans/s. In some cases, movement of the embryo caused motion artifacts to appear in the Doppler images while structural images were at times ruined by brightness artifacts. In both cases, a rescan of the vessel was required and acquisition times were as long as 30 min. The postprocessing time of each data point was ∼2.5  min. Improvements to DS-Eval workflow could be realized by parallelizing the Doppler and structural OCT processing algorithms and upgrading supporting technology, primarily the devices used to maneuver the optical payload into position as this was the most time-consuming process.

Most clinical energy sealing devices use mechanical compression.^32^ Since contactless energy sealing was implemented in this study, it is important to address the potential influences of compression on the overall DS-Eval methodology. First, compressing the vessels before imaging would essentially eliminate the 3-mm vessel diameter limitation posed by the limited optical penetration depth of the imaging beam. The use of DS-Eval could therefore be extended to larger vessels. Second, compression of the vessel would negate the utility of the lemniscate biomarker and many of the other structural biomarkers listed in Table 1. Irradiation outcomes would therefore be more-so dependent on Doppler biomarkers rather than structural biomarkers. The primary biomarker in compression based energy sealing used to delineate seals and nonseals would likely be the presence or absence of flow in the post-Doppler image ROI. Finally, since mechanical compression of the vessel would likely result in a temporary cessation of blood flow, postirradiation scans would have to be conducted once the compression has been released to ensure that flow has ceased due to vessel denaturation. Clinically available systems are not only capable of sealing individual vessels but are also able to seal vascular tissue bundles. In these cases, DS-Eval could still be utilized provided that the lateral width of the imaging window (defined by the galvo sweep range and the distance from focal lens to sample) could be made sufficiently wide to capture all vessels within the bundle. In compression-based approaches, it is assumed that the width of the bundle postirradiation would be significantly less than its width in the natural state. This meaning that the width of the imaging window may not have to be significantly increased in order to apply DS-Eval to vascular tissue bundles.

In this study, it was presumed that DS-Eval had an accuracy of 100%. While this is certainly not absolute, the authors believe that the accuracy of DOCT is very near to the gold standard CT angiography given DOCT’s high velocity resolution. Of course, there are several possible sources of error that could have potentially decreased the accuracy of DS-Eval such as user error (inability to detect failed scans resulting from motion and brightness artifacts) and inherent DOCT limitations. For example, the flow sensitivity of DOCT is related to the A-line rate and the Doppler angle, thus if there was a case where the vessel was scanned in such a way that the imaging beam was orthogonal to the flow direction DS-Eval would fail. This could be avoided by proper optical payload positioning relative to the vessel. In addition, it is also possible to encounter residual flows that are slower than the velocity resolution of the DOCT system, which would also result in a failed classification.

OCT used in this manner has also revealed a variety of biologically related insights into the energy sealing of vessels. Not only was it possible to determine whether the vessel had been sealed, but it was also possible to infer why. For example, by referring to Fig. 4B, it is evident that this vessel did not seal. Further, by observing both the structural and Doppler images, it can be concluded that the vessel did not seal because insufficient thermal energy reached the entirety of the vessel lumen. This conclusion is drawn since the superficial portion of the vessel seems to have been affected while the deeper portion was not. Based on this observation, perhaps vessel sealing using the ${\mathrm{Tm}}^{3+}$ laser could be improved by adjusting the focus of the beam into the center of the vessel lumen. In general, these *in situ* microscopic observations of vascular biomechanical response to laser irradiation are indispensable as they lend greater insight as to the mechanisms involved in laser sealing allowing for improvements to be made to laser sealing systems.

It was found that the outcomes seal or no seal and the evaluation method with which they were determined are dependent and this finding was statistically significant. Referring to Table 3, it can be seen that 64% of FPs reported by V-Eval were partial seals. Subsequently, the specificity of V-Eval was found to be strongly dependent on the type of nonseal classification and this finding was also statistically significant. Therefore, it is reasonable to conclude that in laser sealing studies where V-Eval was used to evaluate energy seals, reported seal rates are likely inflated. Further, this inflation is likely a result of an inability to differentiate partial seals from seals by eye. Meticulous hemostasis is paramount for all surgical procedures, and thus all coagulated vessels should be completely sealed. Partial seals would pose a significant risk, especially if partial seals resulted in slow ooze from the vessel that is not detected visually at the time of surgery. Delayed postsurgical hematomas are not uncommon, and it is reasonable to assume these may be due to incompletely sealed vessels.^33^ In intracranial surgeries, the percentage of postsurgical hematomas requiring surgical intervention ranges from 0.8% to 6.9%.^34^^,^^35^ Thus, current laser technologies used for the purpose of vessel sealing, which were evaluated visually should be reevaluated to ensure that complete vessel seals were achieved.

DS-Eval of vessel seals is not limited solely to laser-based systems, it can be extended for use across all energy sealing systems increasing its utility. Its limitation imposed by a limited penetration depth is compensated for by its high velocity resolution and valuable structural feedback. Further, DS-Eval can be used in the seal classification of large vessels if used intravascularly, which is already a commonly used technique for the evaluation of blood clots.^36^ Here, DS-Eval was presented as a benchtop testing method for laser-based energy sealing systems. Clinical translation of this methodology will not be possible without significant improvement to supporting technology, validation of DS-Eval through comparison with an existing methodology such as CT angiography, and overall modification of biomarkers to suit human anatomy. The system should be modified such that the sample arm is fitted into a handheld contact-based probe similar to existing clinical ultrasound systems. This would potentially reduce acquisition times by up to 15 min since payload maneuvering was the most time-consuming process. In the literature, B-scan postprocessing rates have been reported to be as fast as 40 FPS when GPU-FPGA integrated systems were used.^37^ If these frame rates were implemented here, then the postprocessing times could be decreased from 2.5 min to 5 s. Based on these improvements to supporting technology, it is estimated that the overall acquisition and processing time for a single data point could be performed in under a minute. The primary biomarker used for energy seal evaluation here was the characteristic lemniscate that defined the natural state of the CAM vessel. This lemniscate is not common to human anatomy and thus a new biomarker defining the natural state of human vasculature must be defined. Further, human vessel structure is not constant across all vessel types. For example, arteries proximate to the heart are elastic and feature thinner walls with larger lumina, whereas arteries distal from the heart are muscular with smaller lumina and thicker walls.^38^ Therefore, for clinical translation of DS-Eval, it may be necessary to redefine the natural state biomarker for several vessel types.

## 6 Conclusion

An objective methodology employing both Doppler and structural OCT feedback was introduced for the purposes of energy seal evaluation. The importance of introducing such a methodology is for both evaluating the hemostatic potential of energy sealing systems and for the noninvasive confirmation of hemostasis. Although acquisition and processing times reported here are quite lengthy, this was primarily a feasibility study; clinical translation of this technology can be realized by improving processing speeds and overall ease of use. In general, DS-Eval has the potential to be an indispensable method for the evaluation of energy seals not solely due to its high velocity resolution but also due to valuable microscopic morphological insight regarding the biological mechanisms involved in energy sealing. Future studies will involve dynamic structural and Doppler imaging during laser irradiation to visualize the sealing process.
