# Doppler optical coherence tomography for energy seal evaluation and comparison to visual evaluation (Erratum)

**DOI:** 10.1117/1.JBO.25.4.049801

**Published:** 2020-04-15

**Authors:** Andrew J. Marques, Robnier Reyes, Christopher R. Pasarikovski, Chaoliang Chen, Joel Ramjist, Xijia Gu, Victor Yang

**Affiliations:** a Ryerson University, Bioengineering and Biophotonics Laboratory, Department of Electrical, Computer, and Biomedical Engineering, Toronto, Ontario, Canada; b University of Toronto, Division of Neurosurgery, Department of Surgery, Toronto, Ontario, Canada; c Ryerson University, Department of Electrical, Computer, and Biomedical Engineering, Toronto, Ontario, Canada; d Sunnybrook Health and Sciences Center, Division of Neurosurgery, Toronto, Ontario, Canada; e University of Toronto, Division of Neurosurgery, Faculty of Medicine, Toronto Ontario, Canada

## Abstract

The erratum corrects a misspelled author surname.

This article [J. Biomed. Optics, 25(3), 035003 (2020), doi: 10.1117/1.JBO.25.3.035003] was originally published online on 9 March 2020 with an error in the spelling of an author’s surname. The correct spelling is Pasarikovski.

This article was corrected on 9 April 2020.

